# Association between Blood Lead Levels and Age-Related Macular Degeneration

**DOI:** 10.1371/journal.pone.0134338

**Published:** 2015-08-07

**Authors:** Ho Sik Hwang, Seung Bum Lee, Donghyun Jee

**Affiliations:** 1 Department of Ophthalmology, Chuncheon Sacred Heart Hospital, College of Medicine, Hallym University, Chuncheon, Korea; 2 Seoul St. Mary's Eye Hospital, Suwon, Korea; 3 Department of Ophthalmology and Visual Science, St. Vincent’s Hospital, College of Medicine, Catholic University of Korea, Suwon, Korea; Massachusetts Eye & Ear Infirmary, Harvard Medical School, UNITED STATES

## Abstract

**Purpose:**

To investigate the association between blood lead levels and prevalence of age-related macular degeneration (AMD).

**Methods:**

A nationwide population-based cross-sectional study included 4,933 subjects aged over 40 years who participated in the 2008–2012 Korean National Health and Nutrition Examination Survey, and for whom fundus photographs were available. All participants underwent a standardized interview, evaluation of blood lead concentration, and a comprehensive ophthalmic examination. Digital fundus photographs (45°) were taken of both eyes under physiological mydriasis. All fundus photographs were graded using an international classification and grading system.

**Results:**

Mean blood lead levels were 3.15 μg/dL in men and 2.27 μg/dL in women (*P* < 0.001). After adjusting for potential confounders including age, gender, smoking status, total cholesterol levels, triglyceride levels, heart problems and strokes, the adjusted odds ratio (OR) in women for any AMD was 1.86 (95% Confidence Interval [CI], 1.03–3.36) and for early AMD was 1.92 (95% CI, 1.06–3.48), for those in the highest quintile of lead level compared with the lowest quintile. In men, however, blood lead level was not significantly associated with AMD.

**Conclusions:**

Blood lead levels were higher in men, but were only associated with AMD in women. Increased levels of blood lead may be involved in the pathogenesis of AMD development in women.

## Introduction

Age-related macular degeneration (AMD) is a leading cause of blindness among the elderly in industrialized countries [1]. Although the precise etiology of the condition remains unclear, AMD is known to be a multifactorial disease, involving interactions between genetic and environmental factors [2]. Established risk factors include age and smoking [1, 3]. Other potential factors include cardiovascular disease, dietary oxidant intake, and sunlight exposure, all of which have been inconsistently associated with prevalence of AMD [4–7]. We previously reported that AMD is associated with age, hypertension, and male gender in a representative Korean population [8, 9]. Recently, increasing evidence has suggested that trace metals may play a role in the pathogenesis of AMD [10–12]. For example, we reported that blood cadmium levels were positively associated with prevalence of AMD in a representative Korean population [13].

Lead is a non-essential metal that is toxic to human tissue at very low concentrations [14] and is ranked second most toxic substance in the hazardous substances list by the Agency for Toxicity and Disease Registry [15]. The body burden of lead increases with age, despite efforts to reduce exposure to the metal. The principal sources of lead are paints, water, food, dust, soil, kitchen utensils, and leaded gasoline. Most cases of lead poisoning are attributable to oral ingestion and absorption through the gut [16]. Most ingested lead accumulates in specific target tissues including blood, soft tissues, and bone, where it has a very long half-life [17].

Chronic lead exposure can adversely affect the central nervous, renal, cardiovascular, reproductive, and hematological systems, and can trigger cognitive decline [18–22]. Lead can promote aging by increasing oxidative stress and stimulating production of inflammatory cytokines [23]. In the eye, increased lead exposure is associated with the development of age-related cataracts in men [24] and low-tension glaucoma in women [25]. The retina is particularly susceptible to oxidative stress because of its elevated oxygen tension, high polyunsaturated lipid content, and high level of exposure to light. Very low concentrations of lead can cause detrimental effects to the retina [16, 26–28]. A recent study of 30 autopsy eyes found that lead accumulates in the retinal pigment epithelium (RPE) and choroid [11]. Moreover, excess lead was found in the neural retinas of donor eyes with AMD [29], suggesting that lead accumulation may be associated with the development of AMD. However, most previous studies involve experimental animal studies or case–control studies on human autopsy eyes. Epidemiological studies investigating an association between lead and AMD are limited, and show conflicting results. A recent study examined 5390 participants in the U.S. National Health and Nutrition Examination Survey (NHANES), 2005–2008, and found no association between blood lead levels and AMD [30]. Conversely, the Korean NHANES, 2008–2011, showed a significant association between lead levels and AMD [31]. There is increasing evidence that the detrimental health effects of toxic metals differ in prevalence, or manifest differently, between men and women [32]. Experimental studies suggest that females are more susceptible to the immunotoxic effects of lead [33, 34], thus indiscrimination of blood lead levels by gender could bias the association between lead levels and AMD [35]. Therefore, we evaluated the effect of blood lead levels on AMD prevalence, and additionally analyzed potential effect modification, using data collected from a population-based epidemiological study.

## Subjects and Methods

### Study Population

**Fig 1:**
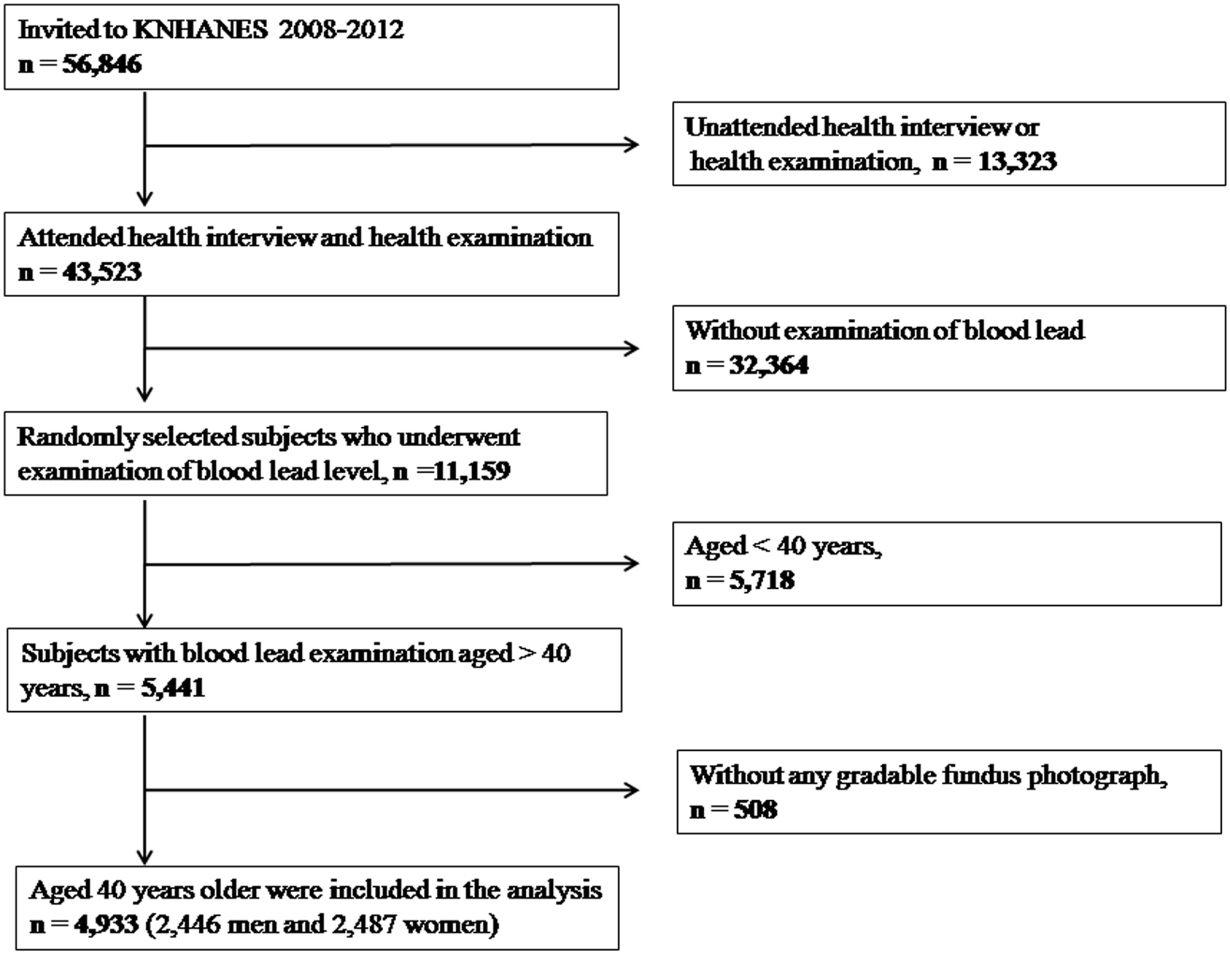
Flow diagram showing selection of study participants.

The present study used data from the Korean National Health and Nutrition Examination Survey (KNHANES). This is an ongoing, nationwide, population-based, cross-sectional survey of nationally representative Korean participants, conducted by the Division of Chronic Disease Surveillance, Korean Center for Disease Control and Prevention. The survey consists of a health interview, a nutritional survey, and a health examination. Details on the study design and methods have been reported previously [36, 37]. Briefly, the KNHANES adopted a rolling sampling design which is a stratified, complex, multistage, probability cluster survey with proportional allocation based on the National Census Registry for the non-institutional civilian population of Korea. Data from the fourth (KNHANES IV, 2008–2009) and fifth (KNHANES V, 2010–2012) surveys were used to investigate the association between blood lead levels and AMD. In the current study, 11,159 individuals whose blood lead levels were measured were selected. Of these, 5,718 who were aged under 40 years, and 508 who did not undergo retinal fundus examinations were excluded. Thus, 4,933 participants aged 40 years or older were included in the analysis (Fig 1). All participants were informed of the aims of the study, and all gave written informed consent. The study design followed the tenets of the Declaration of Helsinki for biomedical research, and was approved by the Institutional Review Board of the Catholic University of Korea in Seoul, Korea.

### Data collection

Digital fundus images were taken under physiological mydriasis using a digital fundus camera (TRC-NW6S; Topcon, Tokyo, Japan). For each participant, a 45° digital retinal image centered on the fovea was taken of each eye (two images per subject). Each fundus photograph was graded twice [38, 39]. Preliminary grading was done at the scene of photography by a trained ophthalmologist, using the International Age-related Maculopathy Epidemiological Study Group grading system [40]. Detailed grading was performed later by 9 retina specialists experienced in grading early and late AMD, who were masked to the patients' characteristics and entrusted by the Korean Ophthalmologic Society (KOS). Final grading was based on the detailed grading, and any discrepancies between the preliminary and detailed grading were resolved by 1 reading specialist. The inter-rater reliability for AMD grading between the preliminary and detailed grading in right and left eyes was 90.2% and 90.7% in 2008, 92.4% and 93.3% in 2009, 94.1% and 95.0% in 2010, 96.2% and 96.6% in 2011 and 96.0% and 96.2% in 2012, (available at: https://knhanes.cdc.go.kr/knhanes/sub04/sub04_03_02.do?classType ¼8).

Early-AMD was defined by the presence of soft, indistinct, or reticular drusen; any type of drusen plus hyper- or hypo-pigmentary changes to the RPE in the macula; or by the presence of soft drusen without late-AMD signs in the macula [38, 39]. Late-AMD was defined by the presence of one of the following lesions: detachment of the RPE or neurosensory retina, hemorrhages in the subretinal or sub-RPE space, disciform scar, or geographic atrophy as a discrete depigmented area with visible choroidal vessels [38, 39]. For subjects with AMD lesions in only one eye, or asymmetric AMD lesions in both eyes, the worst eye was considered.

Demographic information was obtained from health interview data. Height was measured using a wall-mounted measuring scale, and weight was measured using calibrated electronic scales. Body mass index (BMI) values were calculated as follows: weight (kg)/height (m)^2^. Subjects were assigned to age bands of 10 years. Smoking status was self-reported as current smoker, past-smoker, and never-smoker. Alcohol use was self-reported as ever-drinker or never-drinker, wherein never-drinker represented people who had never consumed alcohol during their entire life.

Blood samples were collected after 10–12 h of fasting. Levels of fasting glucose, hemoglobin A1c, total cholesterol, and triglycerides were measured using a Hitachi automatic analyzer (model 7600, Hitachi, Tokyo, Japan). Blood lead levels were determined by graphite furnace atomic absorption spectrophotometry (SpectrAA-800; Varian, Australia). Detection limit was approximately 0.12 μg/L. Details of the lead analysis method have been reported previously [41–43]. All blood analyses were performed in the Neodin Medical Institute, a laboratory certified by the Korean Ministry of Health and Welfare. For internal quality assurance and control, commercial standard reference materials were obtained from Bio-Rad (Lyphocheck Whole Blood Metals Control; Hercules, CA). The coefficients of variation were 0.95–4.83% upon analysis of reference samples. In terms of external quality control, the Neodin Medical Institute has passed the German External Quality Assessment Scheme operated by Friedrick Alexander University. The Scheme assesses the reliability of measurement of low concentrations of chemicals. The Neodin Medical Institute is also certified by the Korean Ministry of Labor as a laboratory competent in the analysis of specific chemicals.

Blood pressure was measured in a sitting position with a sphygmomanometer. After three measurements at 5-min intervals, the average of the second and third measurements was included in the analysis. Diabetes mellitus was defined by a fasting blood glucose level of ≥126 mg/dL or if the individual was taking anti-glycemic medication. Hypertension was defined by a systolic blood pressure of ≥140 mmHg, diastolic blood pressure ≥90 mmHg, or if the individual was taking anti-hypertensive medication. Heart problems were defined as a history of myocardial infarction, and/or angina, and stroke was self-reported.

### Statistical analyses

Statistical analyses were performed using the Statistical Package for the Social Sciences (SPSS) version 18.0 software (SPSS; IBM Corp, Armonk, NY, USA). Strata, sampling units, and sampling weights were used to obtain unbiased point estimates and robust linearized standard errors. Participant characteristics are presented as means and standard errors for continuous variables, percentages and standard errors for categorical variables, and are presented in relation to the presence of AMD. Analysis of variance (ANOVA) or chi-squared testing was used, as appropriate, to compare demographic characteristics.

To evaluate the effect of blood lead level on AMD prevalence, lead levels were grouped into quintiles [44]. Simple and multiple logistic regression analyses were used to explore associations between blood lead level and AMD. After calculation of crude odds ratios (Model 1), we adjusted for age, gender, and other confounders that have been established as AMD risk factors in previous studies, including smoking status, hypertension, lipid profiles, and cardiovascular disease (Model 2) [3, 6, 8]. We evaluated an effect modification by gender by including interaction terms lead and gender in model 2. Blood lead levels were dichotomized at the median concentrations and adjusted odds ratios for low and high lead levels were calculated for each stratum of gender. We also performed separate regression models stratified by gender. All variables in logistic regression analyses were examined in terms of multicollinearity, and only variables with variance inflation factors of less than 10 were used. All *P*-values were two-tailed, and *P* < 0.05 was considered to indicate statistical significance.

## Results

Of 5,441 eligible subjects aged over 40 years for whom blood lead levels were available, the fundus (both eyes) was examined in 4,933 (90.6%) subjects. Reasons for the absence of fundus photographs were as follows: small pupils (42.3%), cataracts (26.7%), poor cooperation (7.1%), refusal (4.8%), corneal opacity (4.4%), and miscellaneous (14.3%). Thus, 4,933 subjects were included in our final analysis and their demographic characteristics are summarized by AMD status in Table 1. Subjects with AMD were more likely to be older (*P* < 0.001), to be never-drinkers (*P*<0.001), and to have higher systolic blood pressure (*P* = 0.031) and hypertension (*P* < 0.001) compared to those without AMD.

**Table 1:** Demographic and clinical characteristics according to early- and late- age-related macular degeneration (AMD) status, and participant status, as reported by the Korean National Health and Nutrition Examination Survey 2008–2012.

Characteristics	No AMD (n = 4,621)	Early-AMD (n = 295)	Late-AMD (n = 17)	*P* ^*a*^	Participants (n = 4,933)	Without fundus photo (n = 508)	*P* ^*b*^
**Male (%)**	55.8 (0.6)	47.9 (3.6)	63.9 (13.5)	.095	55.5 (0.5)	59.4 (3.3)	.251
**Age (yrs)**	51.9 (0.1)	61.2 (0.6)	62.2 (2.0)	<.001*	52.4 (0.1)	59.4 (0.7)	<.001*
**Body mass index (kg/m** ^**2**^ **)**	24.1 (0.1)	23.9 (0.2)	24.9 (0.6)	.258	24.1 (0.1)	24.1 (0.2)	.998
**Systolic blood pressure (mmHg)**	121.7 (0.3)	125.4 (1.3)	131.6 (4.5)	.031*	121.9 (0.3)	125.4 (1.1)	.002*
**Diastolic blood pressure (mmHg)**	79.6 (0.2)	78.7 (0.7)	83.0 (2.7)	.218	79.5 (0.2)	78.4 (0.7)	.203
**Fasting glucose (mg/dL)**	100.9 (0.5)	101.6 (1.2)	103.2 (2.8)	.433	100.9 (0.4)	106.3 (1.9)	.008
**HbA1c (%)**	5.9 (0.0)	5.9 (0.1)	5.7 (0.1)	.045*	5.9 (0.0)	6.3 (0.1)	.001*
**Total cholesterol (mg/dL)**	193.7 (0.6)	193.1 (2.3)	184.4 (9.5)	.330	193.6 (0.6)	190.9 (2.3)	.239
**Triglyceride (mg/dL)**	155.2 (2.7)	141.6 (6.7)	155.7 (24.5)	.818	154.5 (2.6)	162.9 (7.8)	.307
**Lead (μg/dL)**	2.70 (0.03)	2.82 (0.07)	3.23 (0.30)	.080	2.71 (0.03)	2.69 (0.08)	.678
**Diabetes (%)**	11.1 (0.6)	12.1 (2.3)	1.5 (1.6)	.291	11.1 (0.6)	20.3 (2.8)	<.001*
**Hypertension (%)**	36.6 (0.9)	51.5 (3.4)	49.7 (14.5)	<.001*	37.3 (0.9)	52.1 (3.2)	<.001*
**Heart problems (%)**	2.4 (0.3)	4.1 (1.3)	1.3 (1.3)	.143	2.5 (0.3)	3.1 (0.8)	.391
**Stroke (%)**	1.9 (0.2)	2.4 (1.2)	2.8 (2.8)	.762	1.9 (0.2)	3.3 (1.0)	.067
**Smoking status**				.132			.742
Current (%)	33.5 (0.8)	30.3 (3.1)	27.5 (13.0)		33.3 (0.8)	36.5 (3.7)	
Former(%)	18.3 (0.7)	12.6 (2.2)	26.2 (13.2)		18.0 (0.7)	17.4 (2.9)	
Never (%)	48.2 (0.7)	57.1 (3.5)	46.3 (14.3)		48.6 (0.7)	46.1 (3.6)	
**Alcohol consumption (%)**	88.6 (0.5)	78.2 (2.9)	69.3 (13.0)	<.001*	88.0 (0.5)	81.6 (2.7)	.009
**Economic status**				.995			.210
1st quartile (lowest)	27.6 (0.9)	26.5 (3.1)	27.6 (12.3)		34.6 (4.5)	27.3 (0.9)	
2st quartile	26.0 (0.8)	24.9 (3.3)	27.7 (13.1)		25.0 (4.0)	26.0 (0.8)	
3st quartile	23.6 (0.8)	25.7 (3.2)	18.8 (11.9)		24.1 (4.0)	23.7 (0.8)	
4st quartile	22.8 (0.8)	23.0 (3.0)	25.8 (12.6)		16.4 (3.3)	23.0 (0.8)	

Data are expressed as weighted means or weighted frequency (%) with standard errors.

*P < 0.05

***P*** ^***a***^ values compared patients with any AMD and without AMD.

***P*** ^***b***^ value compared patients with and without fundus photograph available.

Demographic and clinical characteristics per quintile blood lead level are shown in Table 2. Subjects with higher blood lead levels were more likely to be male (*P* for trend, <0.001), older (*P* for trend <0.001) and smokers (*P* for trend, <0.001), and to have high systolic blood pressure (*P* for trend, <0.001), high diastolic blood pressure (*P* for trend, <0.001), total cholesterol (p < 0.001), triglyceride (p < 0.001) and hypertension (*P* for trend, <0.001).

**Table 2:** Demographic and clinical characteristics by quintile blood lead category among representative Korean adults aged 40 years or older included in the study.

Characteristics	Blood lead quintiles (μg/dL)					
	1.75< (n = 994)	1.75–2.25 (n = 986)	2.25–2.73 (n = 978)	2.73–3.38 (n = 987)	3.38> (n = 988)	P for trend
**Male (%)**	25.0 (1.7)	43.0 (2.0)	57.1 (1.8)	68.9 (1.6)	80.0 (1.5)	<.001*
**Age (yrs)**	51.3 (0.1)	51.6 (0.3)	52.3 (0.3)	53.1 (0.3)	53.4 (0.3)	<.001*
**Body mass index (kg/m** ^**2**^ **)**	24.9 (0.1)	24.1 (0.1)	24.3 (0.1)	24.2 (0.1)	24.1 (0.1)	.094
**Systolic blood pressure (mmHg)**	119.2 (0.6)	120.1 (0.6)	121.5 (0.6)	122.5 (0.6)	126.1 (0.6)	<.001*
**Diastolic blood pressure (mmHg)**	79.5 (0.4)	78.3 (0.5)	79.5 (0.4)	80.5 (0.4)	80.7 (0.4)	<.001*
**Fasting glucose (mg/dL)**	100.8 (1.4)	100.5 (1.0)	99.7 (0.8)	101.8 (0.9)	101.9 (0.8)	.315
**HbA1c (%)**	6.03 (0.07)	5.97 (0.05)	5.93 (0.05)	5.89 (0.04)	5.95 (0.05)	.563
**Total cholesterol (mg/dL)**	187.7 (1.3)	193.6 (1.5)	195.8 (1.4)	196.1 (1.4)	194.4 (1.5)	<.001*
**Triglyceride (mg/dL)**	129.4 (3.6)	149.2 (7.8)	151.1 (4.0)	164.4 (6.3)	175.7 (6.7)	<.001*
**Diabetes (%)**	13.7 (1.4)	12.2 (1.3)	9.0 (1.0)	10.6 (1.2)	10.3 (1.1)	.068
**Hypertension (%)**	31.7 (1.8)	34.1 (1.9)	35.1 (2.0)	39.8 (1.9)	45.0 (1.9)	<.001*
**Heart problems (%)**	2.6 (0.6)	2.0 (0.5)	2.4 (0.5)	3.0 (0.7)	2.2 (0.5)	.737
**Stroke (%)**	1.8 (0.5)	1.5 (0.4)	2.5 (0.6)	1.6 (0.3)	2.1 (0.6)	.544
**Smoking status**						<.001*
** Never (%)**	76.2 (1.7)	57.6 (2.1)	48.3 (1.9)	38.2 (1.9)	26.0 (1.7)	
** Former (%)**	12.3 (1.4)	17.5 (1.5)	17.3 (1.4)	19.4 (1.6)	23.0 (1.8)	
** Current(%)**	11.5 (1.3)	24.9 (1.8)	34.4 (1.9)	42.4 (2.0)	51.0 (2.0)	
**Alcohol consumption (%)**	81.1 (1.4)	85.4 (1.2)	87.7 (1.2)	90.7 (1.0)	94.5 (0.8)	<.001*
**Economic status**						.303
** 1st quartile (lowest)**	14.6 (1.3)	15.5 (1.3)	17.1 (1.4)	17.7 (1.4)	16.9 (1.3)	
** 2st quartile**	29.9 (1.8)	26.1 (1.8)	24.5 (1.8)	24.9 (1.7)	31.8 (1.9)	
** 3st quartile**	27.9 (1.8)	26.7 (1.8)	28.1 (1.8)	27.7 (1.9)	24.5 (1.5)	
** 4st quartile**	27.6 (1.7)	31.6 (2.0)	30.3 (1.9)	29.6 (1.7)	26.9 (1.8)	

*P < 0.05

**Fig 2:**
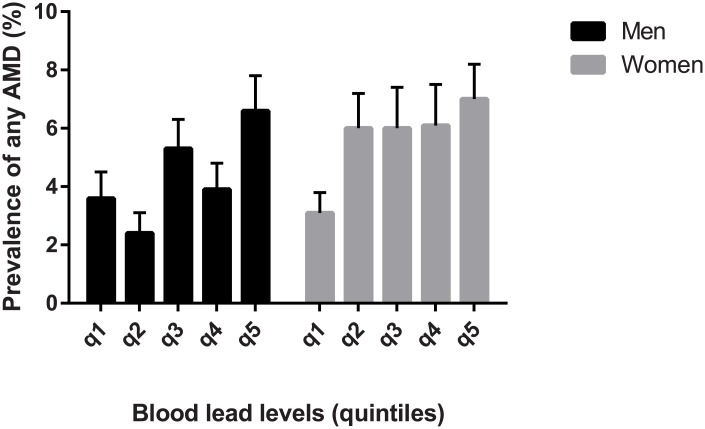
The prevalence of any AMD by quintile of blood lead level, stratified by gender.

Mean blood lead levels were 3.15 μg/dL (95% CI, 3.05–3.25 μg/dL) in men and 2.27 μg/dL (95% CI, 2.22–2.31 μg/dL) in women (*P* < 0.001). In women, mean blood lead levels in subjects with AMD (2.41 μg/dL; 95% CI, 2.27–2.55) was significantly higher than those without AMD (2.23 μg/dL; 95% CI, 2.18–2.27; *P* = 0.013). However, there was no statistically significant difference in blood lead levels between men with and without AMD (3.30 μg/dL; 95% CI, 3.07–3.52 versus 3.07 μg/dL; 95% CI, 3.00–3.15, *P* = 0.069). The prevalence of AMD by quintile of blood lead levels showed a non-linear pattern (Fig 2). The prevalence of AMD increased in the upper 20% of blood lead levels and decreased in the lower 20% of blood levels. However, the prevalence of AMD did not show a significant change in the middle 60% (quintiles 3, 4, and 5) of blood levels.

**Fig 3:**
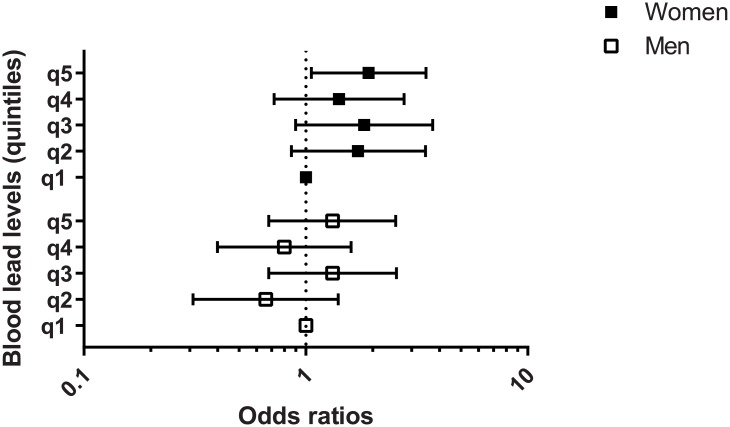
The odds ratio of any AMD by quintile of blood lead level, stratified by gender.

The association between blood lead levels and any type of AMD is shown in Table 3. In women, the adjusted OR for any AMD after adjusting for potential confounders was 1.86 (95% CI, 1.03–3.36) among those in the highest blood lead quintile compared with those in the lowest quintile (*P* for trend = 0.164). In men, however, we found no significant association between blood lead quintile and AMD (Fig 3). The adjusted OR for early AMD in women was 1.92 (95% CI, 1.06–3.48) among those in the highest quintile compared with those in the lowest quintile, but this was without significant linear trend (*P* for trend = 0.159), whereas in men there was no significant association (Table 4). Late AMD was not significantly associated with blood lead level in either gender (Table 5). Using a Wald test for coefficient of interaction term to evaluate effect modification, we found a marginally significant interaction between lead level and any AMD by gender (*P* for interaction = 0.082, Table 6), and between the lead level and late AMD by gender (*P* for interaction = 0.071, Table 6).

**Table 3:** Prevalence and adjusted odds ratio of any type of age-related macular degeneration (AMD), stratified according to quintile category of blood lead among representative Korean adults aged 40 years or older included in the study. Prevalence is expressed as weighted estimates [%] (95% confidence intervals, standard errors [%]).

Blood lead quintiles (μg/dL)	Unweighted number	Prevalence	Model 1	Model 2
**Both genders**				
Quintile 1 (<1.75)	994	4.2 (3.1–5.7, 0.7)	1.00 (reference)	1.00 (reference)
Quintile 2 (1.75–2.25)	986	4.0 (2.9–5.4, 0.6)	0.93 (0.58–1.48)	1.01 (0.61–1.66)
Quintile 3 (2.25–2.73)	978	4.9 (3.5–6.6, 0.8)	1.15 (0.73–1.81)	1.14 (0.70–1.86)
Quintile 4 (2.73–3.38)	987	5.3 (4.0–7.0, 0.7)	1.27 (0.83–1.95)	1.32 (0.83–2.11)
Quintile 5 (>3.38)	988	6.1 (4.7–8.0, 0.8)	1.48 (0.97–2.24)	1.55 (0.96–2.50)
P for trend		.213	.032*	.030*
**Men**				
Quintile 1 (<2.14)	485	3.6 (2.3–5.8, 0.9)	1.00 (reference)	1.00 (reference)
Quintile 2 (2.14–2.64)	493	2.4 (1.3–4.3, 0.7)	0.64 (0.31–1.34)	0.69 (0.33–1.45)
Quintile 3 (2.64–3.12)	488	5.3 (3.6–7.6, 1.0)	1.47 (0.78–2.75)	1.45 (0.76–2.79)
Quintile 4 (3.12–3.79)	485	3.9 (2.5–6.1, 0.9)	1.08 (0.55–2.11)	1.03 (0.51–2.06)
Quintile 5 (>3.79)	495	6.6 (4.6–9.4, 1.2)	1.88 (0.99–3.55)	1.62 (0.86–3.07)
P for trend		.021*	.023*	.090
**Women**				
Quintile 1 (<1.53)	499	3.1 (2.0–4.8, 0.7)	1.00 (reference)	1.00 (reference)
Quintile 2 (1.53–1.94)	504	6.0 (4.1–8.7, 1.2)	1.97 (1.06–3.64)*	1.72 (0.87–3.39)
Quintile 3 (1.94–2.34)	489	6.0 (3.8–9.4, 1.4)	1.99 (1.03–3.84)*	1.73 (0.85–3.51)
Quintile 4 (2.34–2.87)	500	6.1 (3.8–9.6, 1.4)	2.01 (1.03–3.92)*	1.47 (0.73–2.93)
Quintile 5 (>2.87)	495	7.0 (4.9–9.9, 1.2)	2.35 (1.36–4.05)*	1.86 (1.03–3.36)*
P for trend		.141	.011*	.164

Odds Ratios (OR) reflects the risk of AMD in given quintile versus the risk in lowest quintile.

Model 1: Crude OR. Model 2: adjusted for sex, age, smoking, total cholesterol level, triglyceride level, hypertension, heart problem, and stroke.

*P < 0.05

**Table 4:** Prevalence and adjusted odds ratio of early type of age-related macular degeneration (AMD), stratified according to quintile category of blood lead among representative Korean adults aged 40 years or older included in the study. Prevalence is expressed as weighted estimate [%] (95% confidence intervals, standard errors [%]).

Blood lead quintiles (μg/dL)	Unweighted number	Prevalence	Model 1	Model 2
**Both genders**				
Quintile 1 (<1.75)	994	4.0 (2.9–5.5, 0.7)	1.00 (reference)	1.00 (reference)
Quintile 2 (1.75–2.25)	986	3.8 (2.8–5.3, 0.6)	0.95 (0.59–1.54)	1.04 (0.62–1.73)
Quintile 3 (2.25–2.73)	978	4.5 (3.3–6.2, 0.7)	1.13 (0.72–1.78)	1.14 (0.70–1.84)
Quintile 4 (2.73–3.38)	987	4.8 (3.6–6.4, 0.7)	1.20 (0.77–1.86)	1.26 (0.78–2.06)
Quintile 5 (>3.38)	988	5.7 (4.3–7.5, 0.8)	1.44 (0.94–2.22)	1.55 (0.94–2.53)
P for trend		.341	.067	.052
**Men**				
Quintile 1 (<2.14)	485	3.6 (2.3–5.8, 0.9)	1.00 (reference)	1.00 (reference)
Quintile 2 (2.14–2.64)	493	2.3 (1.2–4.2, 0.7)	0.62 (0.29–1.31)	0.66 (0.31–1.40)
Quintile 3 (2.64–3.12)	488	5.0 (3.3–7.3, 1.0)	1.38 (0.73–2.61)	1.32 (0.68–2.56)
Quintile 4 (3.12–3.79)	485	3.2 (2.0–5.1, 0.8)	0.88 (0.44–1.73)	0.80 (0.40–1.60)
Quintile 5 (>3.79)	495	5.8 (4.0–8.5, 1.1)	1.64 (0.86–3.13)	1.32 (0.68–2.54)
P for trend		.044*	.090	.248
**Women**				
Quintile 1 (<1.53)	499	2.9 (1.9–4.6, 0.7)	1.00 (reference)	1.00 (reference)
Quintile 2 (1.53–1.94)	504	5.6 (3.7–8.3, 1.1)	1.94 (1.02–3.69)*	1.72 (0.86–3.46)
Quintile 3 (1.94–2.34)	489	6.0 (3.8–9.4, 1.4)	2.11 (1.08–4.10)*	1.83 (0.90–3.73)
Quintile 4 (2.34–2.87)	500	5.5 (3.5–8.6, 1.3)	1.92 (1.00–3.70)*	1.41 (0.72–2.77)
Quintile 5 (>2.87)	495	6.9 (4.8–9.7, 1.2)	2.42 (1.39–4.22)*	1.92 (1.06–3.48)*
P for trend		.120	.010*	.159

Odds Ratios (OR) reflects the risk of AMD in given quintile versus the risk in the lowest quintile.

Model 1: Crude OR. Model 2: adjusted for sex, age, smoking, total cholesterol level, triglyceride level, hypertension, heart problem, and stroke.

*P < 0.05

**Table 5:** Prevalence and adjusted odds ratio of late type of age-related macular degeneration (AMD), stratified according to quintile category of blood lead among representative Korean adults aged 40 years or older. Prevalence is expressed as weighted estimates [%] (95% confidence intervals, standard errors [%]).

Blood lead quintiles (μg/dL)	Unweighted number	Prevalence	Model 1	Model 2
**Both gender**				
Quintile 1 (<1.75)	994	0.2 (0.1–0.7, 0.1)	1.00 (reference)	1.00 (reference)
Quintile 2 (1.75–2.25)	986	0.1 (0.0–0.4, 0.1)	0.51 (0.08–3.18)	0.53 (0.08–3.35)
Quintile 3 (2.25–2.73)	978	0.3 (0.1–1.3, 0.2)	1.43 (0.22–9.19)	1.30 (0.18–9.27)
Quintile 4 (2.73–3.38)	987	0.6 (0.2–1.6, 0.3)	2.57 (0.52–12.73)	2.34 (0.50–9.27)
Quintile 5 (>3.38)	988	0.5 (0.1–1.4, 0.3)	2.06 (0.40–10.60)	1.62 (0.39–6.77)
P for trend		.485	.115	.113
**Men**				
Quintile 1 (<2.14)	485	0.0 (0.0–0.0, 0.0)	Non applicable	Non applicable
Quintile 2 (2.14–2.64)	493	0.1 (0.0–0.7, 0.1)	1.00 (reference)	1.00 (reference)
Quintile 3 (2.64–3.12)	488	0.3 (0.1–1.4, 0.2)	3.29 (0.28–38.79)	3.00 (0.24–37.24)
Quintile 4 (3.12–3.79)	485	0.7 (0.2–2.7, 0.5)	7.71 (0.71–82.88)	6.89 (0.54–86.87)
Quintile 5 (>3.79)	495	0.8 (0.3–2.6, 0.5)	8.97 (0.92–87.44)	6.27 (0.67–58.42)
P for trend		.111	.003*	.003*
**Women**				
Quintile 1 (<1.53)	499	0.2 (0.0–1.2, 0.2)	1.00 (reference)	1.00 (reference)
Quintile 2 (1.53–1.94)	504	0.4 (0.1–1.4, 0.3)	2.31 (0.23–23.00)	1.79 (0.18–17.51)
Quintile 3 (1.94–2.34)	489	0.0 (0.0–0.0, 0.0)	Non applicable	Non applicable
Quintile 4 (2.34–2.87)	500	0.6 (0.1–3.0, 0.5)	3.44 (0.26–44.47)	2.48 (0.17–36.98)
Quintile 5 (>2.87)	495	0.2 (0.0–1.3, 0.2)	1.04 (0.06–16.83)	2.48 (0.17–36.98)
P for trend		.464	.778	.900

Odds Ratios (OR) reflects the risk of AMD in given quintile versus the risk in lowest quintile.

Model 1: Crude OR. Model 2: adjusted for sex, age, smoking, total cholesterol levels, triglyceride levels, hypertension, heart problem, and stroke.

*P < 0.05

**Table 6:** Effect modification between blood lead levels and age-related macular degeneration (AMD) by gender, age group among representative Korean adults aged 40 years or older included in the study. Association was expressed as odds ratio (OR) with 95% confidence intervals.

	Low blood lead (<2.49 μg/dL)		High blood lead (≥2.49 μg/dL)		P for interaction
	N with/without AMD	OR (95% CI)	N with/without AMD	OR (95% CI)	
**Any AMD**					
Male	37/780	1.00 (reference)	113/1516	1.55 (0.85, 2.83)	
Female	99/1551	1.28 (0.71, 2.30)	63/774	1.61 (1.01, 2.57)	.082
**Early AMD**					
Male	36/781	1.00 (reference)	104/1525	1.43 (0.98–2.31)	
Female	96/1554	1.28 (0.71, 2.30)	60/777	1.47 (0.81, 2.68)	.256
**Late AMD**					
Male	1/816	1.00 (reference)	9/1620	9.89 (1.15, 84.8)	
Female	4/1646	2.20 (0.20, 24.38)	3/834	5.14 (0.41, 64.13)	.071

## Discussion

Our study found that the risk of any AMD and early AMD was significantly increased in women with high blood lead levels compared with those in the lowest quintile of blood lead levels. However, in men there was no significant increased risk of AMD associated with high lead blood levels. Blood lead levels were higher in men (3.15 μg/L) than in women (2.27 μg/L, *P* < 0.001).

We found that higher blood lead levels were significantly associated with decreasing odds of AMD in women, after adjusting for potential confounders. The present study found that the trend analysis for OR did not show a significant ascending trend in AMD as blood lead quintiles per increase. This indicates the possibility of a poor dose-response relationship between blood lead levels and AMD, even though the risk of AMD development is significantly higher in those with high blood lead levels than in those with lower blood lead levels. A recent study using U.S. NHANES data showed no correlation between blood lead levels and AMD [30]. The mean blood lead level in the Korean population studied (2.71 μg/L) was 64.2% higher than those in the U.S. population studied (1.65 μg/L). This suggests that environmental lead exposure may be greater in Korea than in the U.S.A. For example, the regulation of lead use in paint was not instigated in Korea until 1998, while this regulation has been established in the U.S.A since 1978. Another study using Korea NHANES data showed a significant positive association between lead levels and AMD [31]. However, the present study differs from that study in several aspects. First, our study stratified data by gender, while Park et al. showed overall association only. Second, the present study performed logistic regression analysis using raw data, while the previous study used logarithmic transformed data. Finally, the sample size in our study was 4,933 subjects, which is 21.6% larger than in the previous study (3,865 subjects).

Potential biochemical mechanisms of lead-induced predisposition to AMD include oxidative stress and inflammation [45]. The presence of lead in human tissues causes the production of inflammatory cytokines, and increases oxidative stress levels, causing oxidative damage to retinal cells. Lead can increase the production of reactive oxygen species such as the hydroxyl radical, superoxide radical, and hydrogen peroxide. Lead exposure results in lipid peroxidation, DNA damage, and depletion of cell antioxidant defense systems. For example, lead has a high affinity for the sulfhydryl group (SH), and binds to the SH group of glutathione (GSH) which is major cellular antioxidant. Furthermore, oxidative stress and inflammation have been related to the pathogenesis of AMD [46].

In women, the risk of any and early AMD was 1.86- and 1.92-fold higher, respectively, for those in the highest blood lead quintile compared with those in the lowest. However, in men, no significant association between blood lead levels and AMD was shown. A recent study of 98 primary open angle glaucoma patients and 215 controls showed that lead accumulation levels in hair were significantly higher in the female glaucoma patient group compared to the control group, but not between the male groups [25]. In the present study, blood lead levels were higher in men than in women. One possible explanation is that as men have higher hematocrits, and lead binds to erythrocytes, this leads to a rise in blood lead levels [47, 48]. Another possible explanation is a gender-related difference in lead metabolism. More than 90% of lead in the body localizes to bone, with an average half-life of 10 years. The release of lead from bone into the blood is slower in premenopausal women than in men [49], and hormone replacement therapy in women may reduce lead release from bone into the blood [50]. In addition, pregnancy increases mobilization of lead from the maternal skeleton [51]. It is well accepted that blood lead levels in women reflect hereditary factors to a considerable extent (about 40%), while in men they mainly (more than 95%) reflect environmental exposure [52]. Therefore, we propose that blood lead levels are influenced by different factors in men and women. The finding that blood lead levels were higher in men, but were only associated with AMD in women is intriguing, particularly taking into account women's lower blood lead levels compared to men. This may reflect a gender difference in the uptake and metabolism of lead, as well as decreased sensitivity to lead in men or increased sensitivity in women. Further studies are needed to identify the factors responsible for this difference, and especially to elucidate the biological mechanisms of lead absorption and regulation thereof by each gender.

Potential confounders included in this analysis were age, smoking status, hypertension status, total cholesterol levels, triglyceride levels, heart problems and stroke. However, alcohol consumption was not included in the final analysis as a confounder for two reasons. First, alcohol consumption was not associated with AMD after adjusting for gender and age. This was addressed in our previous published article reporting the prevalence and associated factors of AMD in Korea using the same KNHANES data as the present study [8]. Second, most studies examining factors associated with AMD have found that alcohol is not associated with AMD.[6, 53–55] Thus, we did not include alcohol consumption in the regression analysis. Instead, we presented alcohol consumption according to AMD status and lead levels.

The major strength of our present study is the relatively large number of participants (*n* = 4,933) and the study design (a systemic, stratified, multistage, clustered, random sampling method). Another strength is the rigorous quality control of ophthalmic examinations of the fundus and measurement of blood lead levels in KNHANES participants. However, our study has several limitations. First, occupational lead exposure was not recorded. It would have been useful to know whether subjects had been, or were currently involved in smelting, welding, mixing of ceramic glazes, or battery manufacture. However, KNHANES data do not include occupational information. Second, lead exposure status was evaluated only by the measurement of lead levels in blood samples, not in bone or soft-tissue samples. Thus, measurements may not accurately reflect chronic exposure status. However, blood lead level is a good indication of lead body burden in populations with low level environmental exposure [41, 43, 56–58]. Thirdly, KNHANES participants who were ineligible for the present study due to non-gradable fundus photographs, were older than participants who did meet inclusion criteria. Since the older population are more likely to have AMD, this may impact our results. Finally, our study was cross sectional in design, therefore it is difficult to infer causality. However, we report an association between blood lead levels and AMD based on existing evidence of the effect of lead on AMD development. Such a differential pattern of blood lead level is unlikely to be caused by AMD.

In conclusion, the present study provides population-based epidemiological data on the association of blood lead levels with AMD in a representative Korean population. We found that blood lead levels were higher in men but were associated with AMD only in women. This result implies a possible gender difference in the uptake and metabolism of lead, as well as a potential gender difference in sensitivity to lead. In addition, blood lead levels in the Korean population were much higher than those in the U.S. population. Further studies are required to evaluate factors that may be responsible for these gender differences, and to elucidate the biological mechanisms of lead metabolism by gender.
